# Strong sub-seasonal wintertime cooling over East Asia and Northern Europe associated with super El Niño events

**DOI:** 10.1038/s41598-017-03977-2

**Published:** 2017-06-19

**Authors:** Xin Geng, Wenjun Zhang, Malte F. Stuecker, Fei-Fei Jin

**Affiliations:** 1 CIC-FEMD/ILCEC, Key Laboratory of Meteorological Disaster of Ministry of Education (KLME), Nanjing University of Information Science and Technology, Nanjing, China; 2 Department of Atmospheric Sciences, University of Washington, Seattle, Washington USA; 3 Cooperative Programs for the Advancement of Earth System Sciences (CPAESS), University Corporation for Atmospheric Research (UCAR), Boulder, Colorado USA; 4 Department of Atmospheric Sciences, SOEST, University of Hawai’i at Mānoa, Honolulu, Hawaii USA

## Abstract

East Asia experienced a record-breaking cold event during the 2015/16 boreal winter, with pronounced impacts on livelihood in the region. We find that this large-scale cold spell can be attributed to the concurrent super El Niño event in the tropical Pacific. Our analysis reveals that all super El Niño winters (1982/83, 1997/98, and 2015/16) were accompanied by a rapid sub-seasonal North Atlantic Oscillation (NAO)/Arctic Oscillation (AO) phase reversal from a positive to a negative state during early January, which was largely caused by the interaction of these super El Niño events with the subtropical jet annual cycle. The NAO/AO phase transition leads to a rapidly strengthened Siberian High, which favors southward intrusions of cold air to East Asia and thus causes severe local cooling. Similar cold spells can also be detected over Northern Europe associated with the fast sub-seasonal NAO/AO phase reversal. Due to the weaker amplitude of the ENSO forcing, these sub-seasonal atmospheric responses cannot be detected for moderate El Niño events. The super El Niño associated sub-seasonal signal of the East Asian and Northern Europe wintertime temperature responses carries important implications for future predictability of regional extreme events.

## Introduction

**Figure 1:**
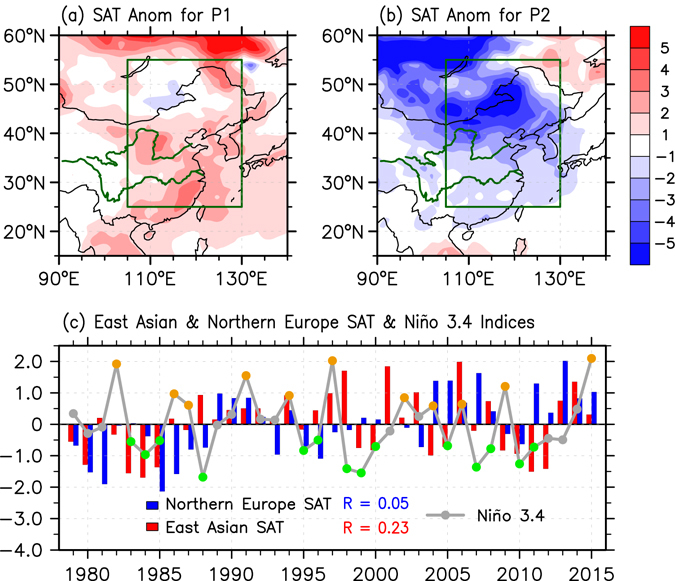
Relationship between ENSO and regional SAT anomalies. Surface air temperature (SAT) anomalies (°C) over East Asia for the period of Dec 19, 2015 to Jan 08, 2016 (P1; **a**) and Jan 12, 2016 to Jan 25, 2016 (P2; **b**). The green box in (**a**) and (**b)** outlines the domain used as the definition of the East Asian SAT index. (**c**) Time evolution of the normalized anomalous East Asian SAT (red bar), Northern Europe SAT (blue bar), and Niño 3.4 (dotted grey curve) indices (°C) during boreal winter (DJF mean) from 1979 to 2015. Here, the winter of 1979 refers to the 1979/80 winter. Orange, green, and grey dots indicate El Niño, La Niña, and normal winters, respectively. Red (Blue) “R” in (**c**) refers to the correlation coefficient between Niño 3.4 and East Asian (Northern Europe) SAT indices. The figure was generated with the NCAR Command Language (Version 6.3.0) [Software] (2015). Boulder, Colorado: UCAR/NCAR/CISL/TDD. http://www.ncl.ucar.edu/.

A cold surge is an energetic and hazardous sub-system of the winter climate in the mid-high latitudes of the Northern Hemisphere^1, 2^. Despite a long-term global warming trend, Eurasia has been suffering from more frequent extreme cold surges in recent years^3, 4^, which often results in heavy snowfall and freezing precipitation with tremendous impacts on human societal and economic activities^5^. During the 2015/16 boreal winter, East Asia first experienced remarkably warmer than normal surface air temperatures (SAT) in the early winter season. However, an extreme cold spell swept subsequently across East Asia during mid-to-late January 2016 (Fig. 1a,b). Similar SAT cooling events can also be observed for Northern Europe (Supplementary Fig. S1). So far, the dynamical mechanisms for these severe wintertime cooling events remain unclear and deserve exploration to improve future predictions of sub-seasonal SAT variations in these regions.

During the 2015/16 boreal winter, we also witnessed a super El Niño event (by the Niño 3.4 measure) in the tropical Pacific, with comparable amplitude to the well-known 1982/83 and 1997/98 extreme El Niño events^6^. It is compelling to hypothesize that this concurrent El Niño event could have played a role in the occurrence of these extreme wintertime cooling events in East Asia and Northern Europe. In fact, great progress has been made in exploring the impacts of the El Niño-Southern Oscillation (ENSO) on seasonal-to-interannual climate variability in East Asia and Europe^7–14^. An anomalous low-level anticyclone is evident over the western North Pacific (WNP) during El Niño winter seasons, which results in the transport of more moist and warm air masses northward to East Asia and thus warmer and wetter than normal winter conditions^7, 13^. Additionally, El Niño tends to cause a negative NAO phase, which results in a colder and drier than normal climate during the late winter season in Europe^8^. Moreover, the ENSO phase is also closely related to the interannual variability of cold surges. For instance, more frequent cold surges occur in East Asia during El Niño winters and less during La Niña winters, due to the modulation of the ENSO associated short-wave train across the North Pacific^15^. However, few studies investigated possible effects of El Niño on the strong sub-seasonal winter climate variations. Especially, the question whether the concurrent super El Niño was responsible for the strong cooling event during the 2015/16 winter is not answered. Here, we explore a possible dynamical linkage between El Niño and strong wintertime SAT cold surge events over East Asia and Northern Europe. Our main finding is that super El Niño winters are generally accompanied by a pronounced North Atlantic Oscillation (NAO)/Arctic Oscillation (AO) phase reversal from a positive to a negative state in early January (not observed during moderate El Niño winters) that gives rise to the severe cooling in both East Asia and Northern Europe in the subsequent mid-to-end January period.

## Results

### Regional wintertime sub-seasonal cooling associated with Super El Niño events

Here we first investigate the relationship between DJF Niño 3.4 and the East Asian SAT indices (see the Methods for details) from 1979 to 2015 (Fig. 1c). Conspicuous interannual variability is displayed in these two indices with a weak in-phase inter-relationship (R = 0.23, insignificant at 90% confidence level). Consistent results can also be detected using de-trended data (not shown). This indicates that although El Niño events tend to be accompanied by a warmer than normal winter in East Asia^7, 13^, this relationship exhibits a large degree of uncertainty. For instance, no prominent warm DJF-mean temperature anomalies were observed in East Asia during two super El Niño events (1982/83 and 2015/16) (Fig. 1c). During the 2015/16 winter, while a pronounced warming occurred during the early winter season, it was followed by a rapid reversal towards an extreme cold surge event (Fig. 1a,b). It can be clearly observed that the East Asian winter climate exhibited a pronounced sub-seasonal transition from an anomalous warm to an anomalous cold state around January 10^th^, 2016 (Supplementary Fig. S2c). Very interestingly, this fast-paced sub-seasonal SAT variability can also be detected during the other two super El Niño events (1982/83 and 1997/98) with only a small difference in the transitional dates (Supplementary Fig. S2a,b). It seems that El Niño events are able to exert a specific modulation on the sub-seasonal evolution of the East Asian SAT, as suggested by theory^16^.

**Figure 2:**
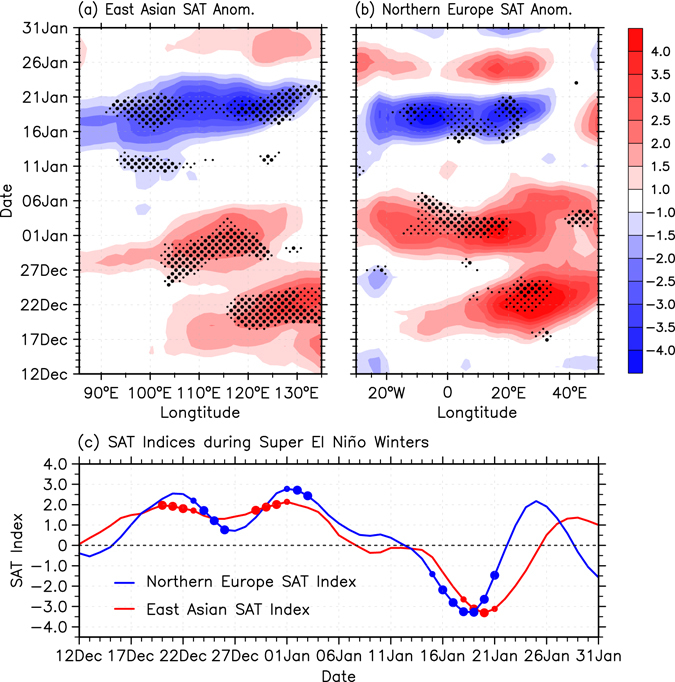
Regional wintertime SAT responses to super El Niño on the sub-seasonal timescale. Composite time-longitude sub-seasonal variation of (**a**) East Asian (25°–55°N average) and (**b**) Northern Europe (60°–80°N average) SAT anomalies (°C) for super El Niño winters (1982/83, 1997/98, and 2015/16). Small and big black dots indicate the anomalies above the 90 and 95% confidence levels, respectively. (**c**) Composite sub-seasonal variability of the East Asian (red line) and Northern Europe (blue line) SAT indices (°C) for super El Niño winters. Small and big dots represent the corresponding SAT anomalies above the 90 and 95% confidence levels, respectively. The figure was generated with the NCAR Command Language (Version 6.3.0) [Software] (2015). Boulder, Colorado: UCAR/NCAR/CISL/TDD. http://www.ncl.ucar.edu/.

Next we utilize a composite analysis for all (both super and moderate) El Niño events to investigate their possible impacts on East Asian sub-seasonal SAT variations. East Asia shows only a very weak and spatially confined warm SAT anomaly in the early winter (between 120° to 135°E during December 21–25, Supplementary Fig. S3a). A similar negligible anomaly is also seen in the East Asian SAT index (Supplementary Fig. S3c). No significant anomalous signal can be observed for the El Niño December and January months in East Asia. Considering the possible uniqueness of super El Niño events with respect to their impacts on East Asian winter climate, we separate them from moderate El Niño events and then repeat our composite analysis. Interestingly, a very different SAT anomaly evolution is observed (Fig. 2a), which is quite similar to that of the 2015/16 winter (Supplementary Fig. S2c). A robust warm anomaly appears east of 100°E in December with anomalous peak temperatures above 3.0 °C for a large meridional average (25°–55°N). This pronounced warm state disappears in the beginning of January when a severe cooling develops in East Asia, attaining a value of −3.5 °C for the same area average. In agreement, the SAT index shows the rapid transition of East Asian SAT from anomalous warm to cold around January 10^th^ (Fig. 2c). The maximum negative SAT value of the index occurs around January 19^th^, close to the time of the lowest climatological temperatures (not shown). The severe cold anomalies superimposed on the lowest climatological SAT can therefore potentially lead to catastrophic consequences.

It is notable that besides in East Asia, the distinct sub-seasonal SAT variation can also be detected in Northern Europe during super El Niño winters. Although the DJF-mean Niño 3.4 index is poorly correlated with the simultaneous Northern Europe SAT index (see the Methods for details; Fig. 1c), super El Niño winters are accompanied by a pronounced SAT warming in the early season and a subsequent strong cooling during the mid-to-late January in Northern Europe (Fig. 2b,c). Moreover, this sub-seasonal cooling signal cannot be detected when moderate El Niño events are considered (Supplementary Fig. S3b,c), thus displaying very similar features to the cold surge events in East Asia.

### Exploration of the modulation pathway

**Figure 3:**
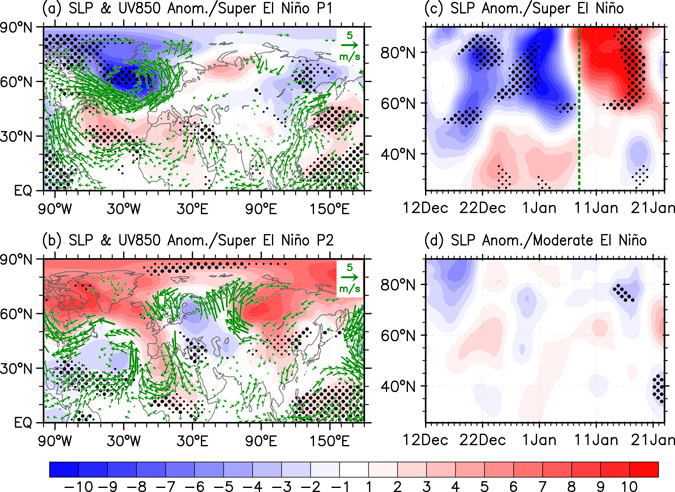
Atmospheric responses to super El Niño on sub-seasonal timescale. Composite SLP (shadings in hPa) and 850-hPa wind anomalies (vector in m/s) for the (**a**) warmer (P1: December 19^th^ to January 8^th^) and (**b**) colder (P2: January 12^th^ to 25^th^) than normal period during super El Niño winters. The wind anomalies are displayed only when they are significant above the 90% confidence level. Composite zonal averaged SLP anomalies (shading in hPa) over the North Atlantic (80°W–30°E) region for (**c**) super and (**d**) moderate El Niño winters. The green dashed line in (**c**) approximately represents the NAO phase transition timing according to the NAO index. Small and big black dots indicate the anomalies above the 90 and 95% confidence levels, respectively. The figure was generated with the NCAR Command Language (Version 6.3.0) [Software] (2015). Boulder, Colorado: UCAR/NCAR/CISL/TDD. http://www.ncl.ucar.edu/.

A scientific question that remains to be answered is why the super El Niño events are accompanied by a severe sub-seasonal SAT phase reversal over East Asia and Northern Europe. To address this question, we first show the anomalous atmospheric circulations for the anomalous warm (P1: early winter) and the following anomalous cold (P2: middle winter) periods (Fig. 3a and b). Here, the warm (P1: December 19^th^ to January 8^th^) and cold (P2: January 12^th^ to 25^th^) periods are defined based on the sub-seasonal phase transition of the SAT indices as shown in Fig. 2c.

During the anomalous warm period (P1) of the super El Niño winter composite, an anomalous low-level anticyclone over the western North Pacific (WNP) and a pronounced positive NAO/AO pattern in the North Atlantic region are evident, which tend to transport more water vapor and warm air from the adjacent ocean towards land, respectively. One can also identify a significantly weakened Siberian High and East Asian trough (Fig. 3a). In contrast, during the cold surge period (P2), the southeastward retreated anomalous WNP anticyclone is drastically confined to the tropics (Fig. 3b). Simultaneously, the Siberian High is prominently amplified both in its intensity and spatial extent. The NAO/AO evidently reverses from a positive (during P1) to a negative phase (during P2). It is notable that we do not distinguish NAO and AO in this study because the NAO is generally regarded as a regional manifestation of the larger scale AO pattern despite some ongoing debates^17–19^. Earlier studies have shown that the NAO is a dominant factor controlling East Asian and Northern European cold surge events^20–23^. In a winter season coinciding with a positive NAO phase, the intense polar vortex and strengthened mid-latitude winds tend to confine the cold polar air to high latitudes. In contrast, during a winter with a negative NAO phase, the weak polar vortex and weakened mid-latitude winds more easily allow southward intrusions of cold polar air southward into mid-latitude regions^24–26^. Over Northern Europe, the negative NAO phase is usually concurrent with weakened Atlantic westerlies, which inhibits warm air transport from the Atlantic Ocean towards Europe. Thus, the NAO phase transition seemingly acts as a bridge to connect super El Niño events and the rapid sub-seasonal transition of winter SAT.

During El Niño winter seasons, one can observe a positive NAO during November–December and a negative NAO during January–March^27, 28^. This sub-seasonally varying ENSO-NAO relationship could be responsible for the weak ENSO-NAO correlation during the boreal winter season mentioned in previous studies^29, 30^. Here, this sub-seasonal atmospheric reversal is further confirmed by the super El Niño composite zonal-mean sea level pressure (SLP) response over the North Atlantic region (Fig. 3c). Significant negative SLP anomalies north of 45°N and positive SLP anomalies to the south are evident during early winter, indicating a positive phase of the NAO. This positive phase persists until early January when the NAO displays a rapid phase reversal around January 8^th^, a couple of days before the SAT cold surge (Fig. 2). In contrast, a similar NAO phase reversal is not observed during moderate El Niño (Fig. 3d), La Niña, or ENSO-neutral winters (Supplementary Fig. S4). Thus, we conclude that the East Asian and Northern Europe cold surge events during super El Niño winters are largely determined by the sub-seasonally varying NAO responses.

### Support from atmospheric model experiments

**Figure 4:**
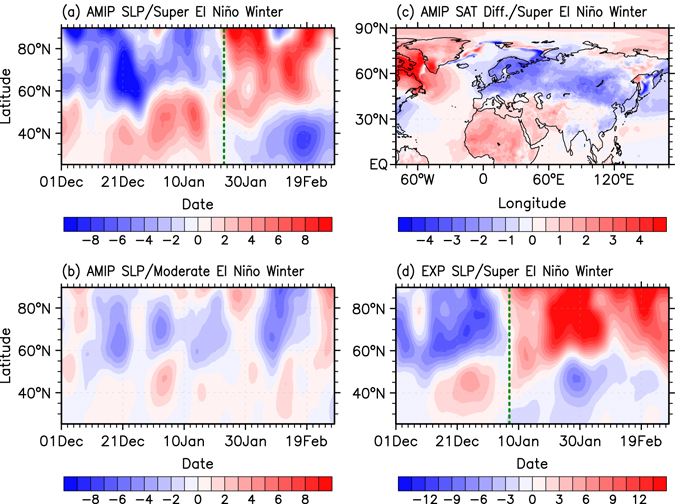
Model simulated responses to El Niño warming on the sub-seasonal timescale. Composite zonal averaged SLP anomalies (hPa) over the North Atlantic (80°W–30°E) region for (**a**) super and (**b**) moderate El Niño winters in GFDL-HIRAM AMIP-style simulations. (**c**) Composite SAT anomalies difference (°C) after and before the sub-seasonal NAO phase transitional date for super El Niño winters in GFDL-HIRAM AMIP-style simulations. (**d**) Zonal average ensemble-mean SLP anomalies (shading in hPa) over the North Atlantic (80°W–30°E) region for the super El Niño winter in the GFDL AM2.1 “Super El Niño” experiment. The green dashed lines in (**a**) and (**d**) approximately represent the NAO phase transition timing according to the NAO index. The figure was generated with the NCAR Command Language (Version 6.3.0) [Software] (2015). Boulder, Colorado: UCAR/NCAR/CISL/TDD. http://www.ncl.ucar.edu/.

We further inspect the manifestation of the aforementioned connection in numerical atmospheric general circulation model (AGCM) simulations. The Geophysical Fluid Dynamics Laboratory global High Resolution Atmospheric Model (GFDL HiRAM, see the Methods for details) is utilized to examine whether the seasonally dependent NAO response during super El Niño winters can be simulated. The experiment is forced with Atmospheric Model Inter-comparison Project (AMIP)-style^31^ sea surface temperature (SST) boundary conditions from 1979 to 2008, a period that includes two super El Niño (1982/83 and 1997/98) and seven moderate El Niño events (1986/87, 1987/88, 1991/92, 1994/95, 2002/03, 2004/05, and 2006/07). The composite zonal-mean SLP sub-seasonal variability over the North Atlantic is displayed for the two super and seven moderate El Niño winters (Fig. 4a,b). The model realistically captures the observed NAO phase reversal signals from positive to negative during the composite super El Niño winter (Fig. 4a) despite a small difference in the exact phase transition date, which occurs approximately at January 23^th^ in the model (slightly later than in the observations). This timing inconsistency may be ascribed to intrinsic model biases and deficiencies. We also examine the anomalous Northern Hemisphere SAT difference after and before the NAO phase transition date during the composite super El Niño winter in the HIRAM model simulations (Fig. 4c). Remarkable cold surge signals are detected for both East Asia and Northern Europe, therefore indicating that this model can well reproduce the sub-seasonal dynamical linkage between super El Niño events, the NAO, and SAT in East Asia and Northern Europe. These results provide some confidence for future dynamical predictability of extreme cold surges associated with super El Niño events. In contrast, neither the NAO phase transition (Fig. 4b) nor the SAT sub-seasonal cooling signal (not shown) can be detected in the composite of moderate El Niño winters, consistent with the observational results.

We further conduct two sets of AGCM experiments using the GFDL Atmospheric Model, version 2.1 (AM2.1) (see the Methods for details) to investigate whether the sub-seasonal SAT anomaly and NAO phase evolution are mainly related to super El Niño-SST over the tropical Pacific. The simulated SLP response over the North Atlantic to super El Niño forcing also realistically reproduces the observed NAO phase reversal signal from positive to negative during the winter season (Fig. 4d). The NAO phase transition date occurs approximately at January 8^th^, exhibiting a similar timing as in the observations. Meanwhile, prominent SAT cooling signals are also detected, with 1.1 °C and 2.4 °C SAT decreases after the NAO phase reversal in East Asia and Northern Europe, respectively (not shown). These results demonstrate a convincing dynamical linkage between super El Niño events, the rapid sub-seasonal phase reversal signals of the NAO, and East Asian and Northern Europe SAT.

### Possible Mechanisms

**Figure 5:**
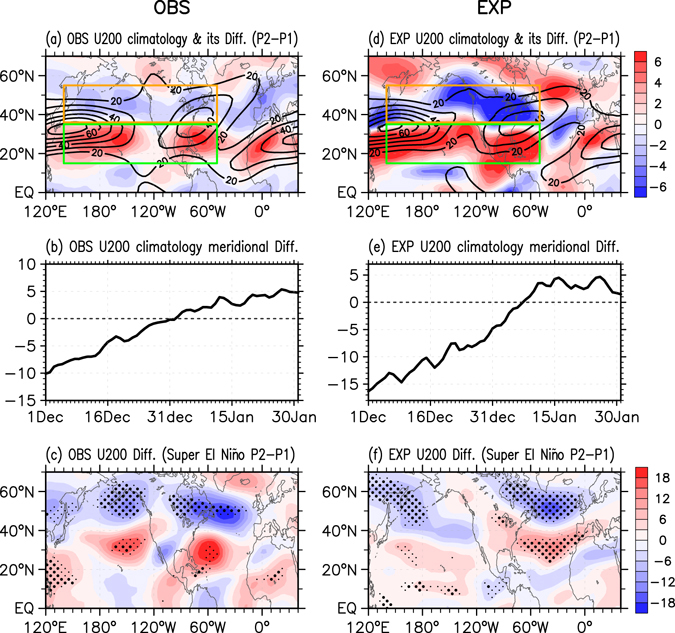
Climatological subtropical jet and its sub-seasonal variations during super El Niño winters. (**a**) 200-hPa zonal wind climatology during P1 and P2 (contours in m/s, from 20 to 70 by 10), and its differences between P2 and P1 (shading in m/s); (**b**) Meridional difference of the 200-hPa zonal wind climatology between the southern (15–35°N, 140°E–50°W, green box) and northern (36–55°N, 140°E–50°W, orange box) parts of the subtropical jet; (**c**) Differences of the 200-hPa zonal wind anomalies (shading in m/s) between super El Niño P2 and P1. (**d–f**) Same as (**a–c**), but for the GFDL AM2.1 model simulations. Small and big black dots indicate the differences above the 90 and 95% confidence levels, respectively. The figure was generated with the NCAR Command Language (Version 6.3.0) [Software] (2015). Boulder, Colorado: UCAR/NCAR/CISL/TDD. http://www.ncl.ucar.edu/.

It now seems evident that the NAO phase transition acts as a bridge to connect the super El Niño events and the rapid sub-seasonal transitions of winter SAT. But by which mechanism super El Niño events induce the rapid NAO phase reversal requires further investigation. Previous studies proposed that the negative NAO responses during El Niño late winters are possibly related to the weakening of the stratospheric polar vortex^32–34^, a modulation by the delayed tropical North Atlantic (TNA) SST warming^35^, or variations of the tropospheric subtropical jet waveguide^36^. Next we investigate these three mechanisms individually. Neither a significant stratospheric downward propagation nor a significant delayed TNA SST warming signal can be detected (Fig. S5). Moreover, with only the tropical Pacific SST anomalies (no TNA SST anomaly) imposed, the GFDL AM2.1 can realistically reproduce the NAO phase reversal (Fig. 4d). These results suggest that the sub-seasonally varying NAO responses could not be attributed to either stratospheric processes or the TNA SST modulation. In contrast, when investigating the 200-hPa zonal winds during the boreal winter season, we find that the climatological jet exhibits a robust southward movement during early January (Fig. 5a). This rapid wind shift can also be clearly shown by the time evolution of the north-south zonal wind difference (Fig. 5b). When a super El Niño event occurs, the climatological southward shift of the jet in P2 may interact with the El Niño in a different way compared to that during P1, which thus could give rise to a different pathway for trapping Rossby waves propagating eastward into the North Atlantic. Correspondingly, the jet anomalies in super El Niño P2 differ from those in P1. The difference (Fig. 5c) shows an evident south-north dipole structure over the Pacific-Atlantic sector, resembling the spatial pattern of climatological jet change (Fig. 5a). It indicates that a significant southward movement of the anomalous jet appears during super Niño early January. Specifically, in P1, the positive zonal wind anomalies are confined near 50°N over the North Atlantic, amplifying the Atlantic jet and thus favoring a positive NAO phase (Supplementary Fig. S6a). In contrast, the positive zonal wind anomalies in P2 are shifted southwards and negative anomalies are evident over the North Atlantic, which corresponds to a negative NAO phase (Supplementary Fig. S6b). The above phenomenon can also be qualitatively demonstrated using the GFDL AM2.1 “Super El Niño” experimental simulations (Fig. 4d and Fig. 5d–f). Thus, the robust southward movement of the climatological subtropical jet possibly plays an important role for the rapid NAO phase reversal under a basically constant SST anomaly forcing in the tropical Pacific. In agreement, the genesis of fast sub-seasonal variability due to an interaction of a slowly varying interannual ENSO signal with the background annual cycle has been demonstrated in a recent theoretical study^16^.

In order to investigate the reason why no significant sub-seasonal NAO phase reversal can be observed during moderate El Niño winters, we next conduct another sets of experiments using the GFDL AM2.1 model (Supplementary Table S1) testing the impact of the El Niño amplitude. As we reduce the “Super El Niño” SST anomalies prescribed in the tropical Pacific, a significant NAO phase reversal cannot be simulated anymore. In agreement with the observations and the AMIP-style simulations (Fig. 3d and Fig. 4b), the “Moderate El Niño” experiment also fails to capture a significant NAO phase transition signal. In contrast, when the “Moderate El Niño” SST anomaly amplitude is increased, attaining the same value as in the “Super El Nino” experiment, the North Atlantic also experiences a significant sub-seasonally paced transition of the NAO response from a positive to a negative phase. Therefore, we conjecture that the observed failure of moderate El Niño driving the sub-seasonal NAO transition is largely ascribed to the weak SST anomaly forcing in the central-eastern tropical Pacific.

## Discussion

The present study demonstrates that super El Niño events tend to be accompanied by a pronounced wintertime SAT warming to cooling transition in East Asia and Northern Europe through a sub-seasonal modulation of the NAO/AO-associated atmospheric circulation. During super El Niño early winters, East Asia and Northern Europe usually experience a warmer than normal winter. However, in the following middle winter season, severe SAT cold surges sweep across these two regions with a rapid SAT drop by about 6 °C over large areas. The different atmospheric circulations during the warm and cold periods suggest that super El Niño events tend to produce a strong sub-seasonal NAO/AO phase reversal from a positive to a negative state through interacting with the climatological southward movement of the subtropical jet during early January. Weakened westerlies, a strengthened Siberian High, and a southeastward retreat of the anomalous WNP anticyclone accompany this phase transition, which results in severe cooling in both Northern Europe and East Asia. Due to the weaker amplitude of the ENSO forcing, the sub-seasonal transition of the NAO/AO phase and the associated winter SAT cannot be clearly detected during moderate El Niño winters.

Using AGCM simulations, the sub-seasonal NAO/AO phase reversal, as well as the cold SAT events over East Asia and Northern Europe, can be realistically reproduced during both the 1982/83 and 1997/98 super El Niño winters. Consistent with observations, simulated moderate El Niño winters do not exhibit the NAO/AO phase transition or the SAT cooling signal. Furthermore, the rapid sub-seasonal NAO/AO phase reversal during super El Niño winters is also well reproduced by our model experiments. Therefore, the super El Niño associated sub-seasonal signal in East Asian and Northern Europe wintertime SAT should aid substantially in the prediction of regional extreme events.

## Methods

### Data

The monthly and daily SST data are obtained from the National Oceanic and Atmospheric Administration (NOAA) Extended Reconstructed SST version 4^37^ (ERSST v4) and NOAA high-resolution blended analysis of Optimum Interpolation SST version 2^38^ (OISST v2) respectively. The 2-metre (T2m) temperatures are utilized to represent the SAT. The monthly and daily SAT and the associated atmospheric circulation variables used to investigate the processes of wintertime cooling events are obtained from the European Centre for Medium-Range Weather Forecasts (ECMWF) Reanalysis (ERA)-Interim dataset^39^. The ERSST v4, OISST v2 and ERA-Interim data are derived from January 1979 to February 2016 and are globally at 2.0° × 2.0°, 0.25° × 0.25° and 1.5° × 1.5° grid resolutions, respectively.

### Methods

Anomalies for all variables were computed as the deviation from the long-time climatological mean. A 5-day moving average filter is performed to exclude the high frequency variability during the boreal winter season when analyzing the daily data. We also used 7-day and 9-day moving average filters and the qualitative results remain unchanged (not shown). We defined an East Asian SAT index as the SAT anomalies averaged over the region of 25°–55°N and 105°–130°E to approximately represent the variability of East Asian SAT. Additionally, a Northern Europe SAT index is calculated as the average of the T2m anomalies over the region of 60°–80°N and 20°W–30°E. The TNA SST index^35^ is defined as an area average of the SST anomalies over 0°–25°N, 80°W–0°. The NAO index is defined by the difference of normalized SLP anomaly between 35°N and 65°N over the North Atlantic sector (zonal averaged from 80°W to 30°E)^40^. An AO index is calculated by subtracting zonal mean SLP at 65°N from zonal mean SLP at 35°N^41^. The daily evolution of SAT during December and January is the focus of this study since the rapid temperature transitions appear in early January. El Niño events are selected based on a threshold of 0.5 °C for the Niño 3.4 (5°S–5°N, 120°–170°W) SST anomaly. We therefore identified 11 El Niño winters (1982/83, 1986/87, 1987/88, 1991/92, 1994/95, 1997/98, 2002/03, 2004/05, 2006/07, 2009/10, and 2015/16). Among them, three events (1982/83, 1997/98, and 2015/16) are classified as super El Niño events since their corresponding Niño 3.4 SST anomalies reach 2 °C during their mature phases. The remaining El Niño events are referred to as moderate El Niño events. All statistical significance tests were performed using the two-tailed Student’s *t* test and the effective degrees of freedom were determined by considering the autocorrelation of the respective time series.

### AGCMs

To verify the connection between super El Niño and regional wintertime cooling events, the AMIP simulations of GFDL HiRAM are used. The model uses a finite-volume dynamical core on a cubed sphere grid with each face consisting of a 180 by 180 grid, with 32 levels in the vertical dimension. The model’s physics parameterizations are similar to GFDL’s AM2.1 model, with modifications deemed appropriate for the increased resolution^42^. We utilize an ensemble average of three simulations with perturbed initial conditions for better reliability.

Moreover, to further confirm the relationship between El Niño forcing and sub-seasonal NAO phase reversal, five sets of experiments are performed based on the GFDL AM2.1, with a horizontal resolution of 2.5° × longitude 2° latitude with specified SST boundary conditions^43^. These experiments are referred to as the “CTRL” (control), “Super El Niño” run, etc. The first set of experiments is forced with a global annual cycle SST while the others have the same annual cycle but with specific El Niño SST anomalies (from the developing year June to decaying year February) added in the tropical Pacific (30°S–30°N, 120°–280°E) (see Supplementary Table S1 for details). Each set of the experiments is run for about 14 months and each consists of twelve ensemble members with different perturbed initial conditions. The differences between the each specific “El Niño” and “CTRL” ensemble means are regarded as the specific El Niño event signal.

## Electronic supplementary material

Supplementary Information
