# Supplementary material for: Strong sub-seasonal wintertime cooling over East Asia and Northern Europe associated with super El Niño events
Source: Sci Rep. 2017 Jun 19;7:3770. doi: 10.1038/s41598-017-03977-2 (PMC5476682; doi:10.1038/s41598-017-03977-2)
Supplement: Supplementary file 1 — Supplementary Information [file 41598_2017_3977_MOESM1_ESM.doc]

Supplementary information for

# Strong sub-seasonal wintertime cooling over East Asia and Northern Europe associated with super El Niño events

Xin Geng^1^, Wenjun Zhang^1*^, Malte F. Stuecker^2,3^ & Fei-Fei Jin^4^

## Contents of this file

Figure S1-S6

Table S1

__________________

^1^CIC-FEMD/ILCEC, Key Laboratory of Meteorological Disaster of Ministry of Education (KLME), Nanjing University of Information Science and Technology, Nanjing, China. ^2^Department of Atmospheric Sciences, University of Washington, Seattle, Washington, USA. ^3^Cooperative Programs for the Advancement of Earth System Sciences (CPAESS), University Corporation for Atmospheric Research (UCAR), Boulder, Colorado, USA. ^4^Department of Atmospheric Sciences, SOEST, University of Hawai‘i at Mānoa, Honolulu, Hawaii, USA.

Corresponding author: Wenjun Zhang (email: zhangwj@nuist.edu.cn).


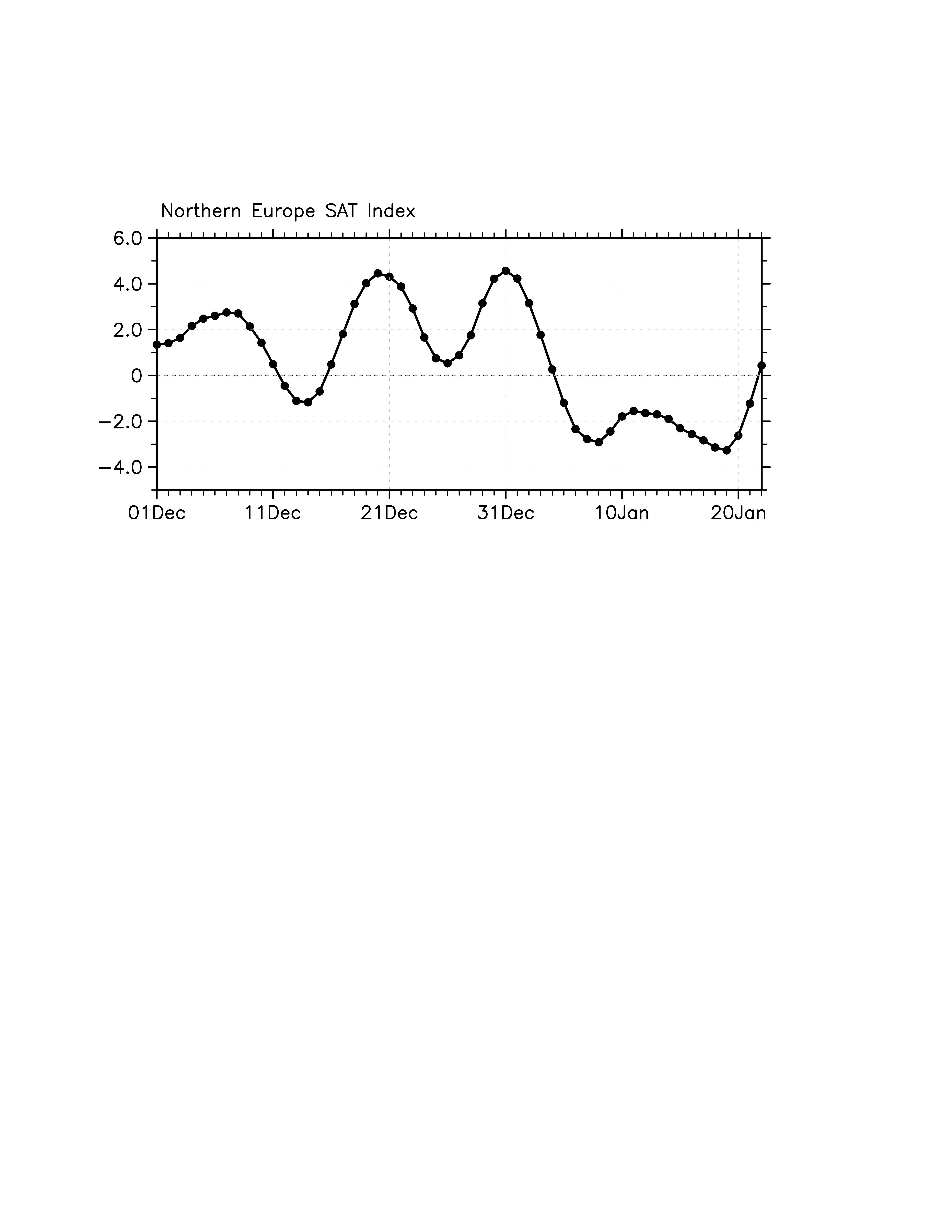


**Figure S1 | Northern Europe anomalous SAT evolution during the 2015/16 winter.** Time evolution of the Northern Europe SAT index (°C) for the 2015/16 super El Niño winter.


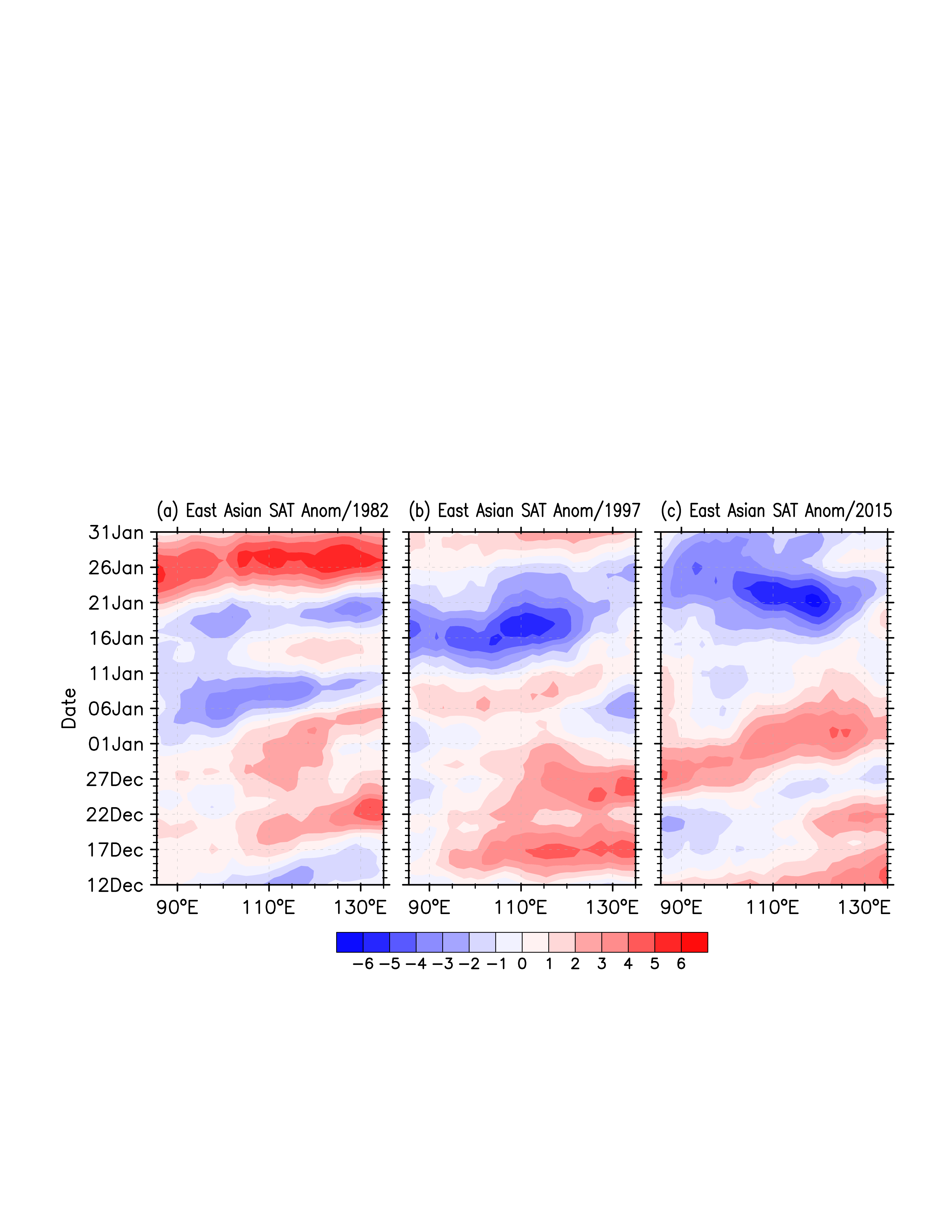


**Figure S2 | East Asian anomalous SAT evolutions during super El Niño winters**. Composite time-longitude East Asian (25°-55°N average) SAT anomalies (°C) for 1982/83 (a), 1997/98 (b), and 2015/16 (c) super El Niño winters.


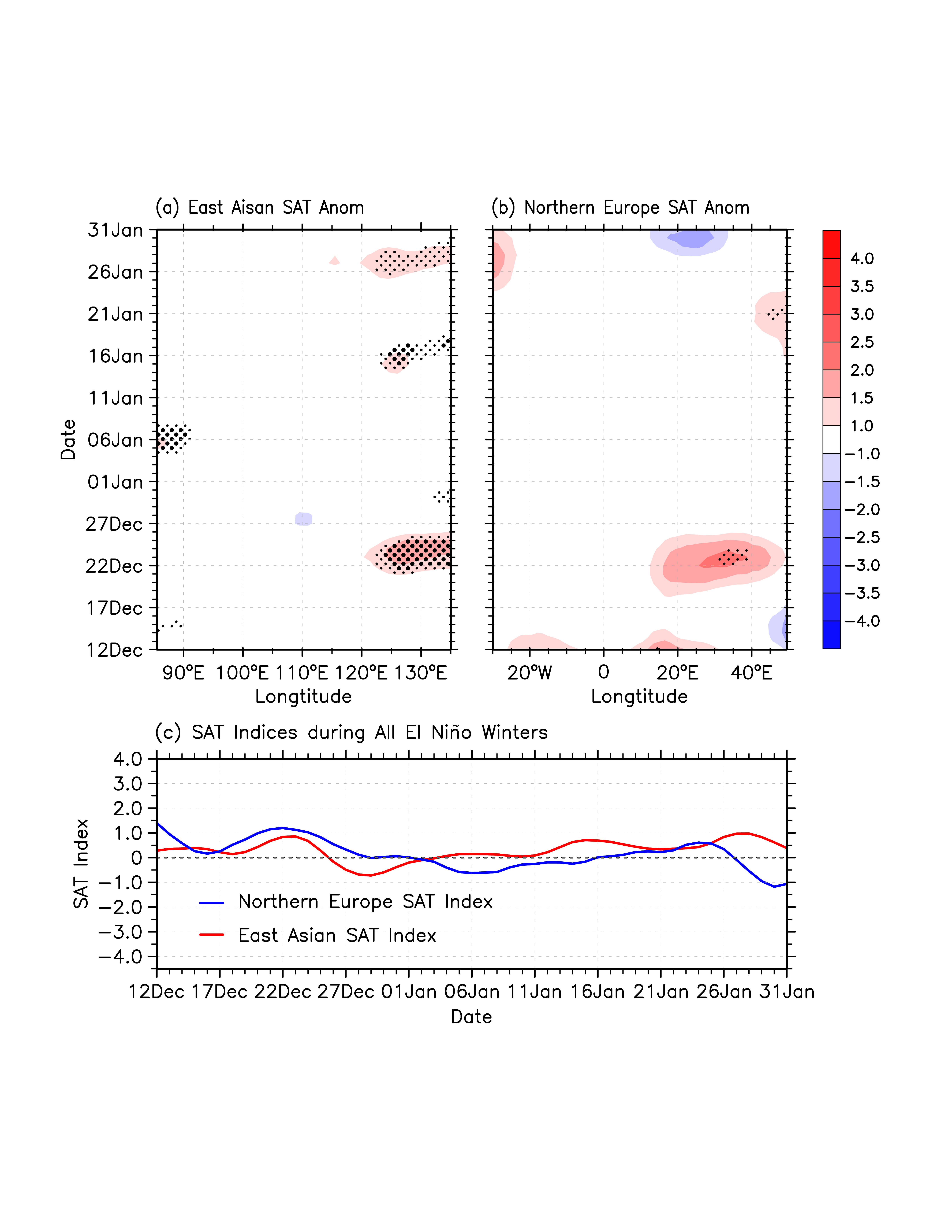


**Figure S3 | Wintertime SAT responses to El Niño on the sub-seasonal timescale**. Composite time-longitude sub-seasonal variation of (a) East Asian (25°-55°N average) and (b) Northern Europe (60°-80°N average) SAT anomalies (shading in °C) for all El Niño winters. Small and big black dots indicate the anomalies above the 90 and 95% confidence levels, respectively. (c) Composite sub-seasonal variability of the East Asian (red line) and Northern Europe (blue line) SAT indices (°C) for all El Niño winters. No significant values are shown based on the 90% confidence level.


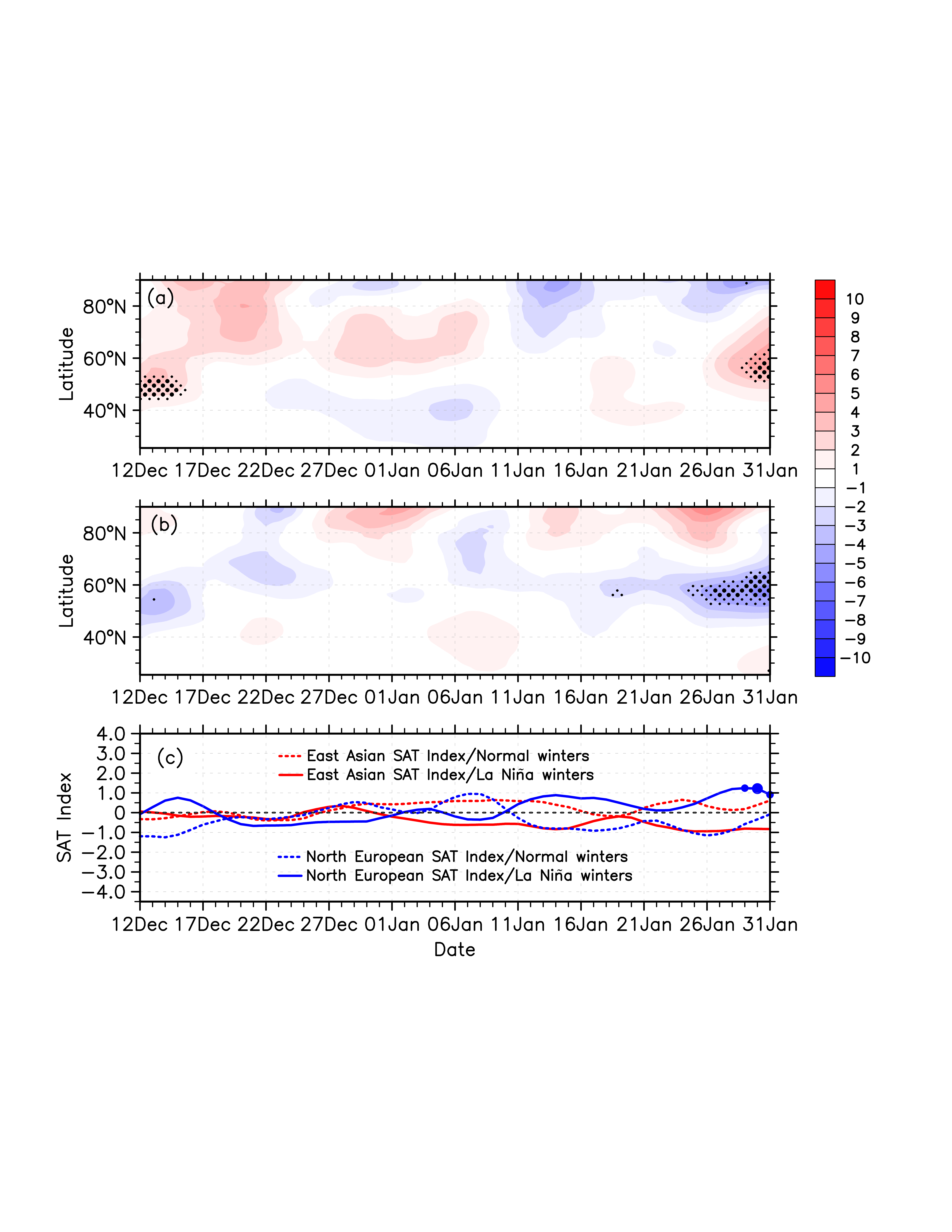


**Figure S4 | Atmospheric and SAT anomalies during La Niña and normal winters**. Composite zonal averaged SLP anomalies (shading in hPa) over the North Atlantic (80°W-30°E) region for (a) La Niña and (b) normal winters. Small and big black dots indicate the anomalies above the 90 and 95% confidence levels, respectively. (c) Composite sub-seasonal variability of the East Asian (red lines) and North European (blue lines) SAT indices (°C) for La Niña (solid line) and ENSO-neutral (dashed line) winters. Small and big dots represent that the corresponding SAT values above the 90 and 95% confidence levels, respectively.


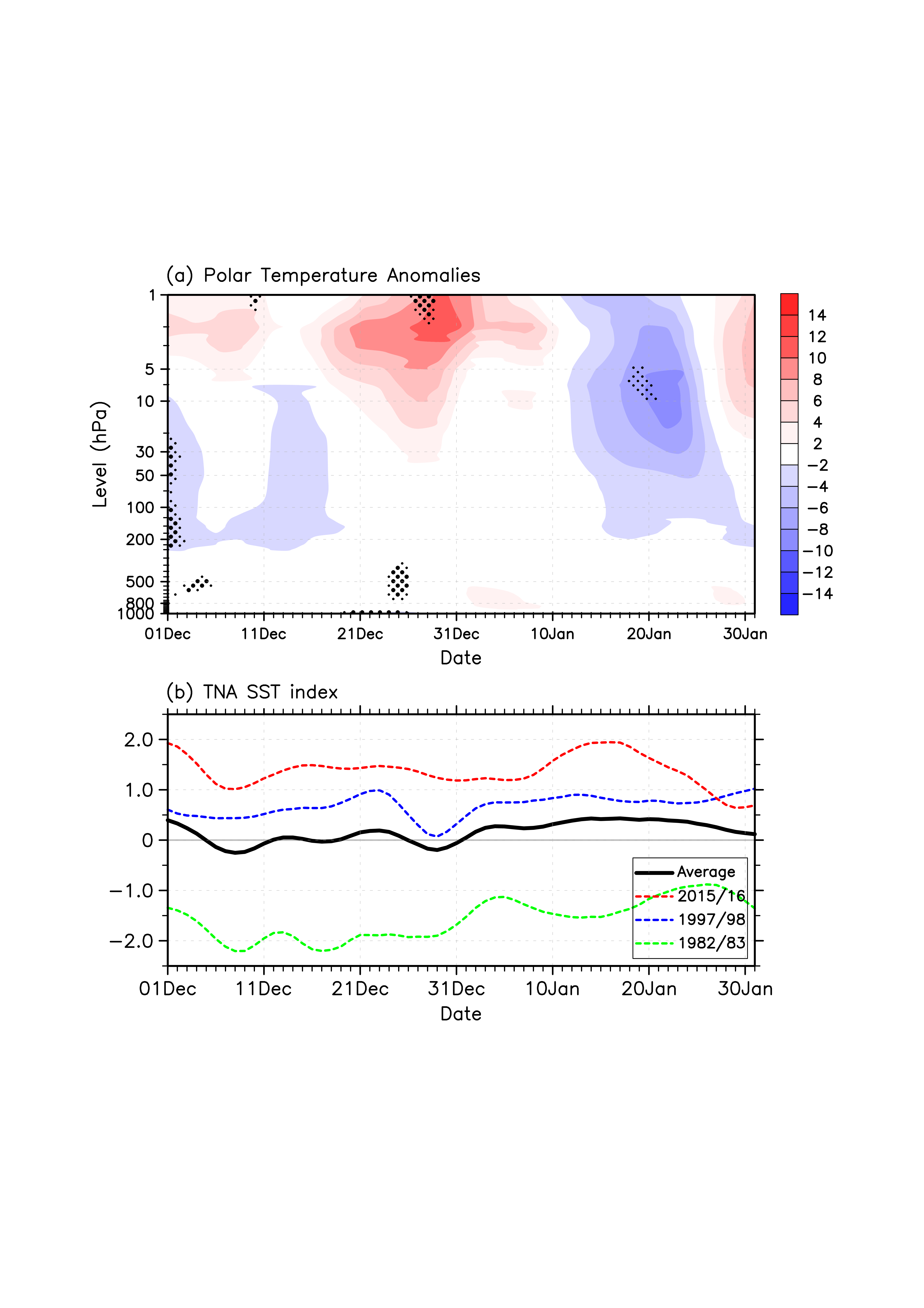


**Figure S5 | Composite vertical temperature anomaly profile and the evolutions of TNA SST index during super El Niño winters. (a)** Time–height evolution of the area-weighted (60°–90°N) zonal mean polar temperature anomalies. Small and big black dots indicate the anomalies above the 90 and 95% confidence levels, respectively; **(b)** Time evolutions of the TNA SST index (°C) for the 1982/83 (green dashed line), 1997/98 (blue dashed line), 2015/16 (red dashed line) super El Niño winters. The black solid line is the composite index of the three super El Niño winters and no significant values are detected based on the 90% confidence level.


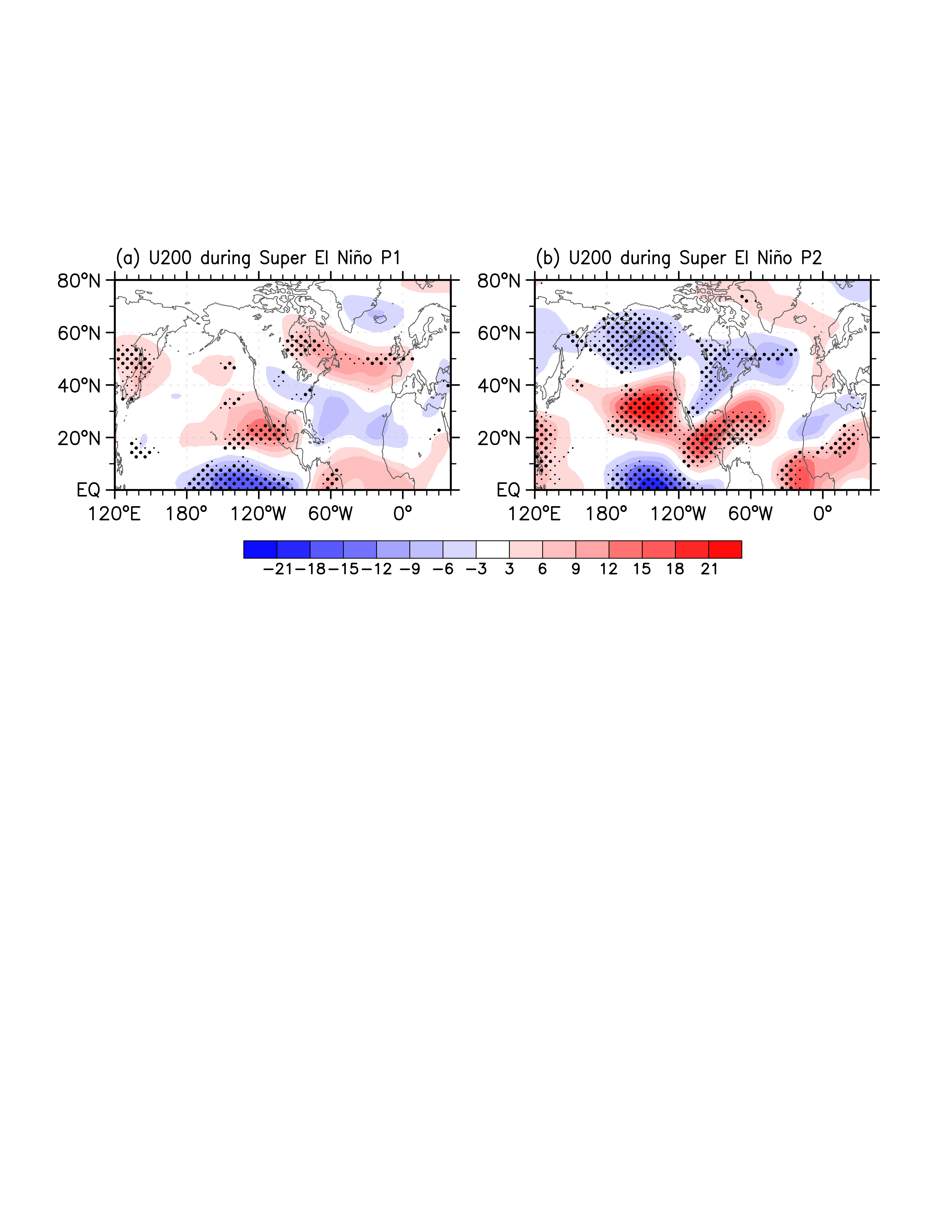


**Figure S6. | Subtropical jet variations during super El Niño winters.** Composite 200-hPa zonal wind anomalies (shading in m/s) for the (a) P1 and (b) P2 during super El Niño winters. Small and big black dots indicate the anomalies above the 90 and 95% confidence levels, respectively.

**Table S1 |** List of the conducted SST perturbation experiments and the resultant NAO differences between P2 and P1. The NAO index in the model simulations is calculated as the difference in regionally zonal-averaged SLP over the North Atlantic sector from 80°W to 30°E between 35°N and 65°N. The blue bold values represent that the differences are significant at the 90% confidence level.

| **Observations or Experiments** | | **Description of the SST perturbation** | **NAO difference (P2-P1)** |
| --- | --- | --- | --- |
| OBS | Super El Niño |  | **-15.82** |
|  | Moderate El Niño |  | -1.53 |
| EXP | Super El Niño | SST anomalies from super El Niño developing year June to decaying year February are imposed in the tropical Pacific (30°S–30°N, 120°E–80°W) | **-16.56** |
|  | Super El Niño_0.5 | As in “Super El Niño” but half intensity of the SST anomalies are imposed | -9.06 |
|  | Super El Niño_0.25 | As in “Super El Niño” but a quarter intensity of the SST anomalies are imposed | -4.16 |
|  | Moderate El Niño | As in “Super El Niño” but for moderate El Niño events | -6.27 |
|  | Moderate El Niño_2.2 | As in “Moderate El Niño” but 2.2 times intensity (the same as “Super El Niño ”) of the SST anomalies are imposed | **-8.66** |
